# Sensor Data Integration: A New Cross-Industry Collaboration to Articulate Value, Define Needs, and Advance a Framework for Best Practices

**DOI:** 10.2196/34493

**Published:** 2021-11-09

**Authors:** Ieuan Clay, Christian Angelopoulos, Anne Lord Bailey, Aaron Blocker, Simona Carini, Rodrigo Carvajal, David Drummond, Kimberly F McManus, Ingrid Oakley-Girvan, Krupal B Patel, Phillip Szepietowski, Jennifer C Goldsack

**Affiliations:** 1 Digital Medicine Society (DiMe) Boston, MA United States; 2 HumanFirst San Fransisco, CA United States; 3 Department of Veterans Affairs Washington, DC United States; 4 Savvy Cooperative Queens, NY United States; 5 University of California San Francisco San Francisco, CA United States; 6 H Lee Moffitt Cancer Center and Research Institute Tampa, FL United States; 7 Evidation Health San Mateo, CA United States; 8 Medable San Fransisco, CA United States

**Keywords:** digital measures, data integration, patient centricity, utility

## Abstract

Data integration, the processes by which data are aggregated, combined, and made available for use, has been key to the development and growth of many technological solutions. In health care, we are experiencing a revolution in the use of sensors to collect data on patient behaviors and experiences. Yet, the potential of this data to transform health outcomes is being held back. Deficits in standards, lexicons, data rights, permissioning, and security have been well documented, less so the cultural adoption of sensor data integration as a priority for large-scale deployment and impact on patient lives. The use and reuse of trustworthy data to make better and faster decisions across drug development and care delivery will require an understanding of all stakeholder needs and best practices to ensure these needs are met. The Digital Medicine Society is launching a new multistakeholder Sensor Data Integration Tour of Duty to address these challenges and more, providing a clear direction on how sensor data can fulfill its potential to enhance patient lives.

## Introduction

Data integration has been defined as “...the technical and business processes used to (aggregate and) combine data from multiple sources to provide a unified, single view of the data” [1]. In practice, this means building bridges across different sources of data, to create an integrated data set that enables you to ask bigger questions or new questions (ie, integration followed by utilization) [2].

Data integration has been a force for innovation and scaling in many technological fields, helping new products become part of our everyday lives. Why do your new headphones work with your phone? Cross-industry collaboration on standards fueled the growth of Bluetooth from its initial invention at Ericsson in the late 1990s to one of the most prevalent current-day technologies [3], incorporated into an estimated 5 billion new devices in 2021 [4]. Love that your credit card works practically everywhere, including internationally? This would not be possible without the Payment Card Industry Data Security Standard (and associated Security Council), which has been a globally accepted standard since 2004 [5]. Trying to get home the fastest way? The apps and services that help us plan and navigate our journeys rely on the General Transit Feed Specification [6], which began in 2005 as a collaboration between Google and the TriMet transport agency and is now a global standard driving the utilization of transit data [7]. All of these examples underline not only the practical impact of successful data integration, but also the critical role of multisector collaboration in achieving success.

Where is the new field of digital health, and sensor data in particular, in its data integration journey and what needs to be done to move it forward?

The Digital Medicine Society (DiMe), a nonprofit organization dedicated to advancing the safe, effective, ethical, and equitable use of digital medicine to optimize health [8], will examine these questions and more as part of a new Sensor Data Integration Tour of Duty. The tour will convene multistakeholder experts from Amazon Web Services, Evidation Health, the Food and Drug Administration (FDA) Center for Drug Evaluation and Research (CDER) and the Center for Devices and Radiological Health (CDRH), Human First, the Institute of Electrical and Electronics Engineers, Medable, the Moffitt Cancer Center, Oracle, Open mHealth, Savvy Cooperative, Takeda, and the United States Department of Veterans Affairs. The Sensor Data Integration Tour of Duty will build off other initiatives like The Playbook [9] in advancing the adoption and utility of digital medicine tools. In this paper, we will describe the current state of sensor data integration in health care, examine the importance of sensor data integration in achieving the promise of digital health to improve clinical research and patient care at scale, and offer a vision of what overcoming these challenges and continuing progress will enable.

## Why Is Sensor Data Integration Important?

In the world of health care, examples of data integration can be found across the drug development and care delivery continuum:

- Modeling and understanding complex disease systems is typically only possible by considering and integrating a wide range of relevant data sources [10,11];
- Data integration supports translating observations from preclinical models to studies with human subjects and is common in early phase drug discovery [12,13];
- Similar data sources combined across different studies create meta-studies to increase statistical power to detect differences and improve the generalizability of results [14,15];
- Bridging clinical to real-world evidence studies aids the translation of observations from clinical development to the market. Examples include converting an outcome used in a clinical trial to deployment to support reimbursement [16] or treatment [17], or comparing error from research or clinical-grade and consumer-grade sensors to better validate large-scale observations [18].

Data integration is ubiquitous in digital health, but the definition above highlights three critical considerations pertaining to sensor data from digital health products:

- Solutions to sensor data integration challenges will be found in both “technical and business processes”;
- “Aggregating and combining data from multiple sources” will provide the critical context for sensor data to drive a learning health care system [19];
- Creating a “unified, single view of the data” will allow the use and reuse of trustworthy data to make better and faster decisions.

Sensor data (ie, data collected by wearable devices, smartphones, and other sensor-equipped connected digital medicine products [20]) is not a silver bullet. We cannot develop new sensor-based measures without combining data from different data sources [21,22], and we cannot deploy those measures without contextualization [23,24].

When we develop a sensor-based digital measure, establishing that it is fit for purpose in a given population involves cross-referencing with clinical and behavioral anchor data to demonstrate accuracy and validity [20,25]. As a therapy moves from clinical development into the hands of patients, it is also critical that clinical outcomes are integrated with real-world data, and that researcher stakeholders and other decision-makers such as payers and clinical policy makers can access and assess those data.

To support utilization and full patient value, health care providers and hospital administrators making treatment decisions all need access to the sensor data and digital measures captured during care and need to be able to digest and infer the same conclusions. Similarly, participants, investigators, statisticians, heads of medical affairs, or regulators need those data to be contextualized and appropriately presented to them, such that they are able to draw insights and value.

Currently, we are far from achieving this potential to improve lives, and it has been claimed that limitations in data integration and utilization are the primary rate limiter [26,27]. Improving lives is always at the front of our minds, making it worthwhile to think about how these tools and applications “look” from the patient’s perspective. Data integration should minimize additional burden caused by management and assessment tools to patients already burdened by their condition(s). Widespread adoption will require interfaces that provide immediate value and are simple, interactive, and intuitive. No longer should patients ask: why does my health care provider not have access to all relevant information? Why should I have to perform the same test in multiple trials, and why do they all have separate apps? I think this data is important; how do I share it with my doctor? Achieving truly integrated continuous care is not possible without effective data integration [28].

## What Are the Key Issues?

There are several specific technical hurdles that get in the way of effective data integration, including the plethora of formats [29,30] and the corresponding lack of consensus around common metadata standards [14,26], although progress is being made [31]. Perhaps more worrisome is the fact that permissioning and consent often exclude integration and exploration [32], and massive gaps in digital health research remain regarding data rights and governance [33,34].

Yet, perhaps the biggest hurdles are cultural adoption, that is, effective incentivization to share data (within and across organizations) and make it easier to integrate [35-37]; recognition that data integration, while perhaps not a glorious pursuit, is central to scaling any digital health tool [38]; and lack of a unified signal from clinical (and other) stakeholders that would enable vendors to address needs around data integration [39,40].

Data integration for digital health, and for sensor data specifically, is in the early phases, and we have yet to see the kind of progress that has been made in other data-heavy health fields (eg, genomics) [41-44]. Currently, no widely adopted data standards or repositories exist, and while progress is being made on the integration of other data types (eg, electronic health records [EHRs], molecular data, patient reported outcomes), we see limited progress around sensor data specifically. This presents an opportunity to provide an early framework for sensor-based data integration and future success of this technology and avoid barriers in integrating other health care data (eg, EHRs). Moreover, understanding the legal and ethical consequences of sharing and integrating sensor data is at a nascent stage [45]. Recent work has shown that “the three most underrepresented areas of research into digital clinical measures were ethics, security, and data rights and governance” [33].

## Where Has Progress Been Made?

Data integration has been recognized as a key driver of value creation in the private sector. In the past few years, we have seen companies invest to improve their data integration abilities through smaller venture capital deals [46], larger acquisitions [47], or internal initiatives like data42 from Novartis [48].

The public space has also been very active, perhaps helped by the inherently precompetitive nature of such work (ie, progress in data integration helps all parties, without any individual party needing to share proprietary data). Across research, clinical development, and in real-world practice, we are starting to see encouraging progress.

Research has been boosted by a number of initiatives, often focused on improving the awareness and availability of digital health data sets. Examples include meta-reviews of publicly available data sets in specific fields to accelerate integration and use of these data, as well as highlighting potential issues and shortcomings like demographic and ethnic underrepresentation [14]. Similarly, new repositories and portals are helping researchers connect and make their data available, such as Synapse and their Digital Health portal [49] or Zenodo [50]. Zenodo is a robust example as it grew out of OpenAIRE [51], a public project focused on enabling open science, demonstrating the concrete progress that specific projects can drive. The COVID-19 Evidence Accelerator [52] has also contributed to integration across the colossal volumes of data being developed as part of the pandemic response.

Clinical development and care delivery are also benefiting from public initiatives around data standards like Substitutable Medical Applications, Reusable Technologies on Fast Healthcare Interoperability Resources (SMART on FHIR) [53], which focuses on EHRs, or the Clinical Data Interchange Standards Consortium, which has had a substantial impact on the submission of evidence to regulatory bodies [54]. RADAR-base (Remote Assessment of Disease and Relapse) [55] grew out of the Innovative Medicine Initiative’s RADAR-CNS (RADAR in Central Nervous System Disorders) [56] and aims to generalize data integration tools and best practices. These initiatives focus both on the integration of a range of devices and data sources, including mobile apps, and the utilization of the data through standardized visualization and analysis tools. Additional examples are the data integration centers in German medical universities, which have been set up to enable use of data across research and care [57]. Establishing these centers is a “meta-collaboration” of 4 consortia funded by the German Ministry of Education and Research. The recently formed Graphite Health is a nonprofit creating solutions for better health system interoperability [58].

Subsequent data utilization efforts bring data rights to the forefront. Who should be able to use the integrated data sets, and for what purpose? How can consenting and integration be structured such that innovation is not hindered and we remain within the ethical boundaries protecting patient rights? The FDA Sentinel Initiative combines claims data from multiple insurers, data from EHRs and patient reports provided by multiple health systems, and even some sensor technologies to evaluate the safety of medical products in the real world [59]. It combines a common data model with a distributed data architecture. Approved users are given secure, structured access to digital health data while respecting privacy and proprietary concerns. The Sentinel Initiative thus protects the data rights of organizations and individuals by keeping data with the holder while still allowing that data to be used for beneficial (eg, research) purposes. Similarly, the SELFIE Horizon 2020 consortium [60] also puts data rights at the forefront, recognizing that data integration is a key pillar of patient-centric, integrated, continuous care, while attempting to balance “preservation of individual privacy and the need for health data sharing” [28].

What does all this mean for sensor data? To achieve real impact on patient lives, sensor data needs to be integrated and contextualized with many other data types. Concerted progress must therefore be made across digital health, and sensor data must keep up with other sources of data like EHRs and break out of condition-specific efforts.

## A Vision for the Future

### Sensor data that is accessible, trustworthy, and relevant

Overcoming data integration challenges is immeasurably worthwhile. If we are to realize the potential of these new data flows to improve patient lives, then we will move toward integrated, holistic, and continuous care [28]. We will provide a smoother, less burdensome, and more valuable experience for patients. We will accelerate research and innovation, and enable the incorporation of data collected from everyday experiences to be included in decision-making. We will dovetail with more mature efforts across health care and do this using a collaborative, ethical, and multisector approach that will ensure that the potential of this data is realized for all stakeholders.

Imagine a situation where a diabetes patient is diagnosed with chronic obstructive pulmonary disease (COPD). The patient can log medication use via an app, and securely and privately funnel activity and sleep data from their user-friendly smartwatch into their doctor’s health care systems. This data is of sufficient quality and accompanied by enough contextual information to facilitate the conversation between patient and doctor (“how have you been feeling?” becomes “I see you have been sleeping better; do you also feel better?’’), and enable better shared decision-making. Exacerbations are tracked and alerts are shared with the patient’s caregivers, which gives the patient peace of mind, and positive reinforcement to further improve this tool. The patient consents that researchers can access their data to improve the detection algorithm, and this data is later reused by a cross-industry consortium to drive qualification of a new COPD outcome.

In oncology settings, the potential is equally appealing. Passive biometric data provides additional data points on a patient’s physical well-being, complementing patient reported outcomes and other data. Worrisome symptoms and activities can be triaged and brought to the clinical care team’s attention. With just-in-time interventions, treatment toxicities can be mitigated or managed early, reducing the downstream costs of readmissions or emergency room visits. Simple interfaces that help patients track their data and symptoms would provide positive reinforcement to support behavior changes. Properly integrated, this data can also help industry partners such as pharmaceutical companies track outcomes and treatment toxicities in clinical trials.

A future where real-world data helps drive the health care system ultimately for preventive actions versus reactive care is a bright one. It is an environment where clinical research and care are not siloed and the data we generate can be referenced again and again, with appropriate consent, to reimagine a system informed by our physiology and how we feel, function, and survive.

### Outlook

Integration and utilization of sensor data as part of the wider landscape of health-relevant data is still in its infancy, but progress is being made. Resolving current shortcomings will reduce burden to, and better serve, all stakeholders: patients, caregivers, clinicians, health care administrators, payers, industry partners, technology manufacturers, analytics companies, and researchers. The Sensor Data Integration Tour of Duty from DiMe includes experts from Amazon Web Services, Evidation Health, the FDA CDER and CDRH, Human First, the Institute of Electrical and Electronics Engineers, Medable, the Moffitt Cancer Center, Oracle, Open mHealth, Savvy Cooperative, Takeda, and the United States Department of Veterans Affairs. Our focus will be to examine stakeholder needs and define a framework for integrating sensor data and making it more useful for decision-making within the wider health care landscape.

Sign up as a DiMe member to stay up to date on findings and resources from this and other projects [8], and check the DiMe e-collection of articles for relevant papers at https://jmir.org/themes/1160-digital-medicine-society-dime.

## Abbreviations

CDER: Center for Drug Evaluation and Research
CDRH: Center for Devices and Radiological Health
COPD: chronic obstructive pulmonary disease
DiMe: Digital Medicine Society
EHR: electronic health record
FDA: Food and Drug Administration
RADAR: Remote Assessment of Disease and Relapse
RADAR-CNS: RADAR in Central Nervous System Disorders
SMART on FHIR: Substitutable Medical Applications, Reusable Technologies on Fast Healthcare Interoperability Resources
